# Functional Identification of AcsR, a MarR Family Transcriptional Regulator Involved in the Regulation of Aromatic Compound-Degrading Genes in *Corynebacterium glutamicum*

**DOI:** 10.3390/microorganisms14040920

**Published:** 2026-04-18

**Authors:** Qimiao Shi, Runge Xu, Meng Shao, Shuli Wang, Ruixue Wang, Jinshuo Liu, Xiaona Li, Ruobing Wang, Ting Zou, Mingfei Yang, Meiru Si, Can Chen

**Affiliations:** 1 Shandong Key Laboratory of Wetland Ecology and Biodiversity Conservation in the Lower Yellow River, College of Life Sciences, Qufu Normal University, Qufu 273165, China; 2 Key Laboratory of Plant Genetics and Molecular Breeding, Henan Key Laboratory of Crop Molecular Breeding & Bioreactor, College of Life Science and Agronomy, Zhoukou Normal University, Zhoukou 466001, China

**Keywords:** *Corynebacterium glutamicum*, AcsR, aromatic compounds resistance, regulator

## Abstract

The MarR (multiple antibiotic resistance regulator) family regulators, which are widely conserved across various organisms, play pivotal roles in metabolism, stress response mechanisms, and virulence factor production. However, the regulatory functions of these factors in the degradation of aromatic compounds within *Corynebacterium glutamicum* remain largely uncharacterized. In this study, we identified a MarR-type regulator, designated AcsR (encoded by *ncgl2425*), which directly represses the expression of the catechol 2,3-dioxygenase gene *ncgl2007* (*c23o*) and the heavy metal (nickel) transport system permease gene *ncgl2351*, while activating the expression of *ncgl2258* encoding an ABC-type C4-dicarboxylate-binding periplasmic protein. AcsR binds specifically as a dimer to a 6 bp inverted repeat sequence, and this binding is disrupted by catechol in vitro. Correspondingly, catechol induces the expression of *c23o* in vivo. Phenotypic analysis revealed that the Δ*acsR* mutant exhibited enhanced resistance to multiple aromatic compounds but increased sensitivity to antibiotics, heavy metals, and oxidants. Collectively, these findings demonstrate that AcsR is an important regulator of stress adaptation in *C. glutamicum* and provide new insights into the regulatory mechanisms of aromatic compound degradation in this industrially important bacterium.

## 1. Introduction

*Corynebacterium glutamicum* is a predominantly aerobic, nonpathogenic, high-G+C, Gram-positive soil bacterium of the *Actinomycetes* family [1]. Over the past six decades, it has been extensively exploited for the industrial-scale production of various L-amino acids and nucleotides.

Notably, *C. glutamicum* ranks among the most arsenic-resistant microorganisms reported to date, and its robust capacity to metabolize aromatic compounds has been well documented [2,3,4,5]. During industrial fermentation, *C. glutamicum* inevitably encounters diverse environmental stresses, including thermal, acidic, high osmotic, and antibiotic stress [6,7]. These stress conditions induce the generation of reactive oxygen species (ROS), leading to oxidative stress [8]. Oxidative stress can be detrimental to cellular integrity, causing damage to key biomolecules [9].

To protect from these stressful conditions during fermentation processes, *C. glutamicum* has evolved a multifaceted protective arsenal, including a thick cell wall enriched with lipoarabinomannan (LAM), mycolic acid, phosphatidylinositol (PI) [10], a millimolar concentration of mycothiol (MSH) [11], protective enzymes such as peroxidase [12,13,14], and stress-responding transcriptional regulators [15,16,17]. Among these defense systems, the ability of transcriptional regulators to directly sense stress signals and initiate rapid responses represents a highly efficient protective strategy.

In *C. glutamicum*, several families of stress-sensing transcription factors have been extensively studied, including the LysR (DNA-binding transcriptional dual-lysine regulator), MarR (multiple antibiotics resistance regulators), TetR (a tetracycline repressor protein), ArsR (As(III)-responsive transcriptional repressors), and XRE (xenobiotic-response element) families [18,19,20,21,22,23,24,25,26]. Of these, the MarR family represents one of the most abundant classes of transcriptional regulators in this bacterium [27]. MarR family members are widely distributed across bacterial species and play crucial roles in adaptation to dynamic and complex environments [18,19,23,24,25,26]. These regulators typically function as negative regulators involved in physiological processes such as catabolism of aromatic compounds, response to environmental stress, and antibiotic resistance [28,29]. Usually, MarR-type regulators bind to the promoter regions of the target genes as homodimers [28,29]. However, specific anionic lipophilic molecule ligands, such as salicylic acid (SA), benzoate (BA), and antibiotics, can alter protein–DNA interactions. MarR-type regulators exhibit ligand promiscuity, capable of binding diverse small molecules [28,30]. Conformational variation in the DNA-binding domain occurs due to ligand binding, releasing the MarR-type regulator from DNA fragments and derepressing target genes. Under certain conditions, the DNA-binding activity of MarR proteins is also regulated by oxidation of cysteine residues under oxidative stress [23,25,26,31,32,33,34]. To date, it remains unclear whether any MarR-type regulators in *C. glutamicum* can respond to multiple signals, such as aromatic compounds and oxidative stress, through distinct molecular mechanisms. Furthermore, genomic analyses reveal that *C. glutamicum* harbors several gene clusters encoding enzymes involved in aromatic compound catabolism and oxidative stress response, suggesting the potential existence of additional, yet uncharacterized MarR homologs that may coordinate responses to both aromatic substrates and oxidative stress response. Therefore, structural and functional characterization of novel MarR family proteins identified through genome mining will enhance our understanding of the diverse stress resistance mechanisms in this industrially important bacterium.

In the genome of *C. glutamicum* RES167, the gene *ncgl2425* encodes a protein containing a helix–turn–helix DNA-binding motif and belongs to the MarR family. In this study, NCgl2425, named AcsR (Aromatic compound-sensing regulator), was found to function as a transcriptional activator of *ncgl2258* (encoding an ABC-type C4-dicarboxylate-binding periplasmic protein), while also functioning as a transcriptional repressor of *ncgl2007* (encoding a catechol 2,3-dioxygenase (C23O)) and *ncgl2351* (encoding a heavy metal (nickel) transport system permease). To our knowledge, this is the first experimental demonstration that MarR-type AcsR from *C. glutamicum* possesses the ability to regulate the degradation of aromatic compounds.

## 2. Materials and Methods

### 2.1. Bacterial Strains and Growth Conditions

Bacterial strains and plasmids used in this study are listed in Table S1. *Escherichia coli* and *C. glutamicum* were grown in Luria–Bertani (LB) media or CGXII minimal medium (CGXII MM) with 2% (*w*/*v*) glucose (Glc) at 37 °C or 30 °C as previously reported [3,14,35]. The *C. glutamicum* RES167 strain (restriction-deficient variant derived from the ATCC 13,032 type) was the parent of all derivatives used in this study. Cellular growth was monitored based on the optical density (OD) at 600 nm. Antibiotics were added at the following concentrations: kanamycin (KAN), 50 µg mL^−1^ for *E. coli* and 25 µg mL^−1^ for *C. glutamicum*; nalidixic acid (NAL), 40 µg mL^−1^ for *C. glutamicum*; and chloramphenicol (CHL), 20 µg mL^−1^ for *E. coli* and 10 µg mL^−1^ for *C. glutamicum*.

### 2.2. Plasmid Construction

The primers used in this study are listed in Table S2. The knockout plasmids pK18*mobsacB*-Δ*acsR* and pK18*mobsacB*-Δ*c23o*, used to construct in-frame deletion mutants, were constructed using gene splicing by overlap extension-PCR (SOE-PCR) [3].

To clone *acsR*, the gene was amplified by PCR from genomic DNA of the *C. glutamicum* RES167 strain using the corresponding primer pairs. These DNA fragments were digested and inserted into pET28a and pXMJ19 to yield pET28a-*acsR* and pXMJ19-*acsR*.

The *lacZY* fusion reporter vectors pK18*mobsacB*-*P_c23o_::lacZY* were constructed as previously described [3].

To obtain pK18*mobsacB-P_c23oM_::lacZY*, a 960 bp *c23o* promoter DNA fragment containing the mutagenized sequence of the AcsR-protected region (*P_c23oM_*) was first constructed by overlap PCR-based site-directed mutagenesis [12].

### 2.3. Overexpression and Purification of Recombinant Protein

The plasmid pET28a-*acsR* was transformed into *E. coli* BL21 for overexpression and purification of His_6_-AcsR. His_6_-AcsR was purified using His·Bind Ni-NTA resin (Novagen, Sigma-Aldrich, St. Louis, MO, USA). The Bradford assay was used to determine protein concentrations [36].

### 2.4. Electrophoretic Mobility Shift Assays (EMSA)

The binding of His_6_-AcsR to the different promoter regions was performed using the method of Wennerhold et al. [37]. Briefly, increasing concentrations of His_6_-AcsR (0–7.5 μg) were incubated with the promoter regions of *ncgl0473*, the ABC-type siderophore-mediated iron acquisition system (SIAS) operon, *c23o*, the *ncgl2259-ncgl2258-ncgl2257-ncgl2256* operon, *ncgl2351*, the *clpS*-*ncgl2428-ncgl2427-ncgl2426-acsR* operon, and *genR* (*ncgl2921*) (final concentration of 40 ng) in a total reaction volume of 20 μL.

EMSA was also performed using Fam 5′-end-labeled promoter DNA probes. Fluorescent FAM-labeled probe Fam-*P_c23o_* (180 bp) or Fam-*P_ncgl2258_* (245 bp) was amplified with Dpx DNA polymerase from *C. glutamicum* genomic DNA with primers E_C23O_-5′Fam-F/E_C23O_-R or E_ncgl2258_-5′Fam-F/E_ncgl2258_-R (Table S2). The Fam-labeled probes were purified by the Wizard^®^ SV Gel and PCR Clean-Up System (Promega, Madison, WI, USA) and were quantified with NanoDrop 2000C (Thermo, Waltham, MA, USA). The unlabeled *P_c23o_* or *P_ncgl2258_* competitor DNA was amplified from *C. glutamicum* genomic DNA with primers E_C23O_-F/E_C23O_-R, or E_ncgl2258_-F/E_ncgl2258_-R. Each 20-μL EMSA reaction solution containing 50 mM Tris-HCl [pH 8.0], 100 mM KCl, 2.5 mM MgCl_2_, 0.2 mM DTT, 2 μg salmon sperm DNA, 10% glycerol, 50 ng Fam-DNA, unlabeled DNA (5 μg) as the competitor, and purified His_6_-AcsR (5 μg) was prepared according to the manufacturer’s protocol (LightShift Chemiluminescent EMSA kit; Thermo Fisher Scientific, Waltham, MA, USA). After reaction solutions were pre-incubated for 30 min at room temperature, the reaction mixtures were subjected to electrophoresis on a 6% native polyacrylamide gel with 5% glycerol in 0.5× TBE electrophoresis buffer, and the DNA promoter was detected with ImageQuant LAS 4000 mini (GE Healthcare, Tokyo, Japan).

The loss of binding due to catechol was tested as follows. Catechol (a final concentration of 15 µM) was added to the binding buffer containing different concentrations of purified His_6_-AcsR and 40 ng 180 bp *P_c23o_* for EMSA. The binding buffer was incubated at room temperature for 30 min and then separated on an 8% nondenaturing polyacrylamide gel, and the gel was stained using SYBR Gold nucleic acid staining solution.

### 2.5. β-Galactosidase Assay

The *lacZY* fusion reporter plasmids pK18*mobsacB*-*P_c23o_::lacZY* and pK18*mobsacB*-*P_c23oM_::lacZY* were transformed into corresponding *C. glutamicum* strains by electroporation. The transformants were selected by plating on LB agar plates containing 40 µg mL^−1^ NAL, 25 µg mL^−1^ KAN, and 10 µg mL^−1^ CHL. The resulting strains were grown in LB medium to an optical density at 600 nm of 0.6–0.7, and then treated with different reagents of various concentrations at 30 °C for 30 min. β-Galactosidase activities were assayed with o-Nitrophenyl-β-D-Galactopyranoside (ONPG) as the substrate [26]. The standard assay for quantifying the amount of β-galactosidase activity in cells, originally described by Miller [26] for the assay of bacterial cultures, involves spectrophotometric measurement of the formation of the yellow chromophore ο-nitrophenol (ONP) as the hydrolytic product of the action of β-galactosidase on the colorless substrate ONPG. All β-Galactosidase experiments were performed with at least three independent biological replicates.

### 2.6. Sensitivity Assays

To evaluate cell viability following stress treatment, dilution and plating on LB plates were performed as previously described [22]. Overnight cultures of *C. glutamicum* strains grown in LB medium at 30 °C were diluted 100-fold with LB medium, and the diluted cells were exposed to different antibiotics, heavy metals, alkylating agents, oxidants, and aromatic compounds at 30 °C with 100 rpm shaking for 30 or 40 min. After treatment, the cultures were subjected to 10,000-fold diluted and plated onto LB agar plates, and colonies were counted after 36 h of growth at 30 °C. Percentage survival was calculated as follows: [(CFU (Colony-Forming Unit) mL^−1^ after challenge at different stresses)/(CFU mL^−1^ before stress challenge)] × 100.

### 2.7. RNA Sequencing (RNA-Seq)

RNA preparation and transcriptome analysis assays followed protocols in prior studies [38]. Total RNA was extracted from the exponentially growing *C. glutamicum* RES167 parental strain and Δ*acsR* mutant (3 biological replicates) via the RNeasy Mini Kit (Qiagen, Hilden, Germany) and the DNase I Kit (Sigma-Aldrich, Taufkirchen, Germany). RNA degradation and contamination were monitored on 1% agarose gels; RNA purity was checked using a NanoPhotometer spectrophotometer (IMPLEN, Westlake Village, CA, USA), and RNA integrity was assessed using a Bioanalyzer 2100 system (Agilent Technologies, Santa Clara, CA, USA). A total of 5 μg RNA per sample was used as input material in RNA sample preparations for subsequent cDNA library construction. All 6 samples had RIN values above 7.0. Sequencing libraries were generated using an Illumina HiSeq^TM^ 2000 RNA Sample Preparation Kit (Illumina, San Diego, CA, USA) following the manufacturer’s recommendations and four index codes were added to attribute sequences to each sample. Differential expression analysis was performed using the NOIseq method (Sonia Tarazona 2100). *p*-values were adjusted using the Benjamini–Hochberg method. A corrected *p*-value of ≤0.01 and a log_2_ (the gene expression ratio of the *C. glutamicum* Δ*acsR* mutant to *C. glutamicum* RES167 parental strain (WT)) of +1.7 or −1.7 were set as the threshold for significantly differential expression. Gene Ontology (GO) enrichment analysis of the differentially expressed genes (DEGs) was implemented by the GOseq R package (version 1.3.1, Haibao Tang, Pasadena, CA, USA), in which the gene length bias was corrected. GO terms with corrected *p*-values less than or equal to 0.01 were considered to indicate significant enrichment of DEGs. RNA-seq data for this study have been deposited in the NCBI SRA database under accession number PRJNA1058395.

### 2.8. Size Exclusion Chromatography

The molecular mass of the purified protein of His_6_-AcsR was estimated as described in our previous publication [39].

### 2.9. DNase I Footprinting Assay

DNase I footprinting assay was performed according to Liu et al. [22].

### 2.10. Quantitative Reverse Transcription-Polymerase Chain Reaction (qRT-PCR) Analysis

Mid-log phase WT(pXMJ19), Δ*acsR*(pXMJ19), and Δ*acsR*(pXMJ19-*acsR*) strains cultivated in LB broth were exposed to different agents at the indicated concentrations for 30 min and then used to isolate total RNA using the RNeasy Mini Kit (Qiagen, Hilden, Germany) and the DNase I Kit (Sigma-Aldrich, Taufkirchen, Germany). Purified RNA was reverse-transcribed with random 9-mer primers and MLV reverse transcriptase (TaKaRa, Dalian, China). Quantitative RT-PCR analysis (7500 Fast Real-Time PCR; Applied Biosystems, Foster City, CA, USA) was performed as described previously [12]. The primers used were listed in Table S2. To obtain standardization of results, the relative abundance of 16S rRNA was used as the internal standard.

### 2.11. Assays for Enzymatic Activity

Catechol 2,3-dioxygenase (C23O) activity was determined as described in a previous publication [40].

### 2.12. Determination of Kinetic Constants of C23O

The kinetic parameters were determined at different concentrations of catechol (0–200 µM).

The enzyme C23O activities were measured in a 50 mM potassium phosphate buffer (pH 7.5), 2 μM C23O, and catechol. The assay was performed as described in our previous publication [41].

### 2.13. Thermal Field Emission Scanning Electron Microscope (SEM) and Transmission Electron Microscopy (TEM) Analysis

For recording scanning electron microscopy images, cells were cultivated in LB medium, centrifuged for 2 min at 8000 rpm and resuspended in PBS, fixed with 2.5% (*w*/*v*) glutaraldehyde for at least 6 h, washed three times in PBS, and dehydrated by incubating consecutively in an ascending ethanol series (30, 50, 70, 90, and 100%) for 15 min each. The samples were further treated with isopentyl acetate for 30 min and centrifuged, then coated with a layer of gold and observed using a Sigma 500 VP SEM (Carl Zeiss AG, Oberkochen, Germany) at 2 kV.

Cell morphology was examined by TEM analysis as described previously [42]. *C. glutamicum* cells harvested at the late-exponential phase were washed twice with 0.1 M phosphate-buffered saline (PBS; pH 7.4), mixed with 1% low-temp gelling agarose, and further cut into 1 mm^3^ dimensioned gel pieces. The samples were prepared according to the standard TEM procedure. For TEM observations, all images were captured using an HT7800 TEM (Hitachi, Tokyo, Japan).

### 2.14. Statistical Analysis

Three biological replicates were conducted for all the experiments. The data are presented as means ± standard deviation (SD) (n = 3). The statistical significance analyses of differences between groups in gene expression data, LacZY activity, survival assay, and growth curve were evaluated using a paired two-tailed Student’s *t*-test. Statistical analyses were performed using GraphPad Prism software 8. *p* < 0.05 was considered statistically significant.

## 3. Results

### 3.1. Response of the acsR Mutants to Stress-Causing Agents

NCgl2425, designated here as AcsR, was categorized through homology analysis as a member of the multiple antibiotic resistance (MarR) family of transcriptional regulators [28,29]. However, the genome-wide profiling of AcsR target genes and its physiological function were not established.

**Figure 1:**
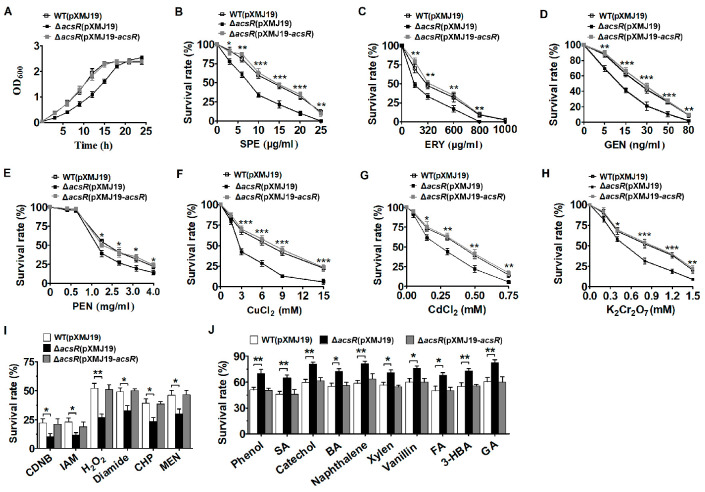
AcsR was involved in stress response. (**A**) Growth of the WT(pXMJ19) strain (the *C. glutamicum* RES167 parental strain (WT) with the empty plasmid pXMJ19), Δ*acsR*(pXMJ19) mutant (the mutant lacking *acsR* with the empty plasmid pXMJ19), and the complementary strain Δ*acsR*(pXMJ19-*acsR*) (the Δ*acsR* mutant expressed the wild-type *acsR* gene with a shuttle vector pXMJ19) in LB broth without stress was used as a control. (**B**–**H**) The indicated three strains grown to the stationary phase in LB broth were exposed to spectinomycin (SPE), erythromycin (ERY), gentamicin (GEN), penicillin (PEN), CuCl_2_, CdCl_2_, or K_2_Cr_2_O_7_ for 40 min, respectively, and the viability of the cells was determined. (**I**) The indicated three strains grown to the stationary phase were exposed to 100 mM 1-Chloro-2, 4-dinitrobenzene (CDNB), 40 mM iodoacetamide (IAM), 100 mM hydrogen peroxide (H_2_O_2_), 5 mM diamide, 5.5 mM cumene hydroperoxide (CHP), and 4 mM menadione (MEN) stress for 30 min at 30 °C, respectively. The viability of the cells was determined. (**J**) The indicated three strains grown to the stationary phase were exposed to 60 mM phenol, 35 mM salicylate (SA), 150 mM catechol, 100 mM benzoic acid (BA), 38 mM naphthalene, 30 mM xylene, 80 mM vanillin, 15 mM ferulic acid (FA), 2 mM 3-hydroxybenzoate (3-HBA) or 10 mM gentisic acid (GA) stress for 30 min at 30 °C, respectively. The viability of the cells was determined. Data showed the averages of three independent experiments, and error bars indicated the SDs from three independent experiments. The asterisk indicated a significant correlation between the WT(pXMJ19) strains and Δ*acsR*(pXMJ19) mutants at *** *p* < 0.001, ** *p* < 0.01, and * *p* < 0.05.

To define the possible involvement of AcsR in defense against stress, an in-frame deletion mutant (Δ*acsR*) was first generated via two-step homologous recombination (Figure S1A) and analyzed with respect to growth pattern and cell morphology. Remarkably, the Δ*acsR*(pXMJ19) mutant (the *acsR* deletion mutant expressing pXMJ19) exhibited a slower exponential growth and decelerated growth rate in LB broth media compared with the WT(pXMJ19) strain (the *C. glutamicum* RES167 parental strain (WT) transformed with the empty plasmid pXMJ19) (Figure 1A). However, the Δ*acsR*(pXMJ19) mutant reached the usual final cell density similar to that of the WT (pXMJ19) strain. SEM and TEM analysis showed that the cell size, cell surface structure, and cell envelope of the Δ*acsR*(pXMJ19) mutant grown on LB broth media had no change compared with the WT strain (Figure S2). These findings led us to hypothesize that AcsR plays a crucial role during growth, and its tightly regulated genes contribute significantly to both growth adaptation and responses to stressors.

Subsequently, we assessed the responses of the Δ*acsR* mutant to various stress-inducing agents. As demonstrated by growth in Figure 1B–H, the tested antibiotics and heavy metals [spectinomycin (SPE), erythromycin (ERY), gentamicin (GEN), penicillin (PEN), Cu^2+^, Cd^2+^, and Cr^6+^] caused a significantly reduced survival rate of the Δ*acsR*(pXMJ19) mutant compared to the WT(pXMJ19) strain. To confirm that the sensitivity to the tested agents was attributed to the absence of the *acsR* gene, the complementary strain Δ*acsR*(pXMJ19-*acsR*) (the Δ*acsR* mutant expressing the wild-type *acsR* gene in the shuttle vector pXMJ19) was constructed, and complementation experiments were performed. As shown in Figure 1B–H, the survival rate of the Δ*acsR*(pXMJ19-*acsR*) strain was significantly improved under stress, which was equivalent to that in the WT(pXMJ19) strains. Together, these results indicated that AcsR was required for antibiotics and metal ions resistance in *C. glutamicum*.

Finally, we examined whether the deletion of *acsR* affected the activity of various alkylating agents, oxidants, and aromatic compounds against three *C. glutamicum* strains: WT(pXMJ19), Δ*acsR*(pXMJ19), and Δ*acsR*(pXMJ19-*acsR*). As shown in Figure 1I, deletion of the *acsR* gene led to increased sensitivity to 1-Chloro-2,4-dinitrobenzene (CDNB), iodoacetamide (IAM), hydrogen peroxide (H_2_O_2_), diamide, cumene hydroperoxide (CHP), and menadione (MEN). However, the sensitivity of the Δ*acsR*(pXMJ19) mutant to various aromatic compounds, including phenol, salicylate (SA), catechol, benzoic acid (BA), naphthalene, xylene, vanillin, ferulic acid (FA), 3-hydroxybenzoate (3-HBA), or gentisic acid (GA), was significantly lower than that of the WT(pXMJ19) or Δ*acsR*(pXMJ19-*acsR*) strains (Figure 1J). These findings showed that AcsR was also involved in resistance to oxidants and aromatic compounds in *C. glutamicum*.

### 3.2. The Impact of the acsR Mutation on Transcription

To clarify the transcriptional changes caused by *acsR* deletion, three RNA-seq-based transcriptomic experiments were performed. In total, 69 genes exhibited at least 3.25-fold alterations in mRNA ratios with *p*-values < 0.05; these are listed in Table S3. Table S3 also includes a few genes that do not meet the criteria but are part of operons containing genes.

**Figure 2:**
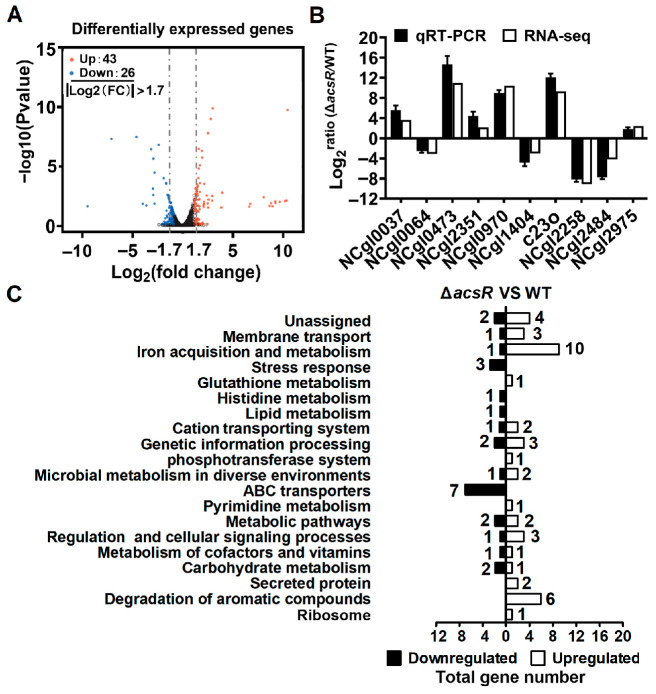
RNA-seq-based transcriptomic analysis of AcsR-regulated genes in *C. glutamicum.* (**A**) Volcano plot of differentially expressed genes. The genes with significant differences were indicated by red (upregulation) and blue dots (downregulation). The *X*-axis represented the log_2_-transformed value of the expression fold changes, and the *Y*-axis indicated the −log_10_-transformed value of statistically significant differences in expression changes. The threshold lines at ±1.7 were labeled with gray dotted lines. (**B**) Validation of RNA-seq data using qRT-qPCR. Ten representative genes were evaluated for validation of the RNA-seq data using qRT-PCR. (**C**) KEGG pathway analysis of differentially expressed genes (Δ*acsR* mutant vs. WT). The black and white bars represented down and upregulated genes, respectively, and the numeric labels represented the number of genes with corresponding functions. All the data went through a log_2_ transformation.

In total, 26 genes showed an mRNA ratio Δ*acsR*/WT of <0.308 (Figure 2A), including those encoding metabolic enzymes (e.g., *ncgl1213*, *ncgl2248*, *ncgl2258*, and *ncgl2399* coding for a L-glyceraldehyde 3-phosphate reductase, isocitrate lyase, ABC-type C4-dicarboxylate binding protein, and gluconate kinase, respectively), two operons for ABC-type phosphonate transporters (the *ncgl2483*-*ncgl2484*-*ncgl2485* and *ncgl1403*-*ncgl1404*-*ncgl1405-ncgl1406*), a transcriptional regulator WhcE (*ncgl0734*), and an alkylhydroperoxidase (*ncgl2286*).

In total, 43 genes were found to have increased mRNA levels in the Δ*acsR* mutant (mRNA ratio Δ*acsR*/WT of >3.25) (Figure 2A), with 12 of them showing increases of 75- to 2500-fold. The genes with strongly increased expression code for a putative transcriptional regulator NCgl0357, a putative cell surface hemin receptor NCgl0377, a membrane protein of unknown function (NCgl0473), a cytosolic siderophore-interacting protein (NCgl0635), two proteins of unknown function (NCgl0970 and NCgl1462), a putative dinucleotide-binding enzyme (NCgl1212), a putative secreted protein (NCgl1651), a glucan phosphorylase (NCgl2006), a putative catechol 2,3-dioxygenase C23O, a malonic semialdehyde reductase (NCgl2913), and an IclR family transcriptional regulator GenR (NCgl2921), respectively.

Next, to validate the results based on the RNA-seq analysis, ten representative genes exhibiting altered mRNA levels in the Δ*acsR* mutant were chosen to perform qRT-PCR, namely, *ncgl0064*, *ncgl1404*, *ncgl2258*, and *ncgl2484* as examples of downregulated genes; and *ncgl0037*, *ncgl0473*, *ncgl0970*, *c23o*, *ncgl2351*, and *ncgl2975* as upregulated genes. The log_2_-transformed values of qRT-PCR were in good agreement with the log_2_-transformed fold changes in RNA-seq-based transcriptomic data (Figure 2B). This result signified that the RNA-seq data were credible.

Finally, the functions of the sixty-nine differentially expressed genes (DEGs) were determined using KEGG pathway analysis. As shown in Figure 2C, 19 pathways were identified from the DEGs, including iron metabolism, aromatic compound degradation, ABC transporters, and the stress response. In combination with the observed phenotype of the Δ*acsR* mutant, the altered expression of many genes indicated that AcsR may be a non-negligible factor in regulating aromatic compound degradation and stress response. Consequently, proteins related to the degradation of aromatic compounds and the stress response garnered our particular interest.

### 3.3. Binding to the Promoter Regions of c23o and the ncgl2259-ncgl2258-ncgl2257-ncgl2256 Operon

RNA-seq-based transcriptomic analysis revealed several putative target genes of AcsR. To determine which genes with strongly differential expression in the RNA-seq experiments were direct target genes of the transcriptional regulator AcsR, EMSAs were performed with purified His_6_-AcsR protein. We analyzed the promoters of six genes that showed more than a 158-fold change in mRNA ratio, but the promoter of *ncg12351* was chosen because it encodes a heavy metal (nickel) transport system permease protein. The promoters were proposed by PROM-Prediction of bacterial promoter and RNA-seq-based newtrans analysis (Figure S3). AcsR was predicted to form an operon with NCgl2426 (rhomboid family protein), NCgl2427 (L-aminopeptidase/D-esterase), NCgl2428 (hypothetical protein), and NCgl2429 (ATP-dependent Clp protease adaptor protein ClpS) by RNA-seq-based newtrans analysis, NCgl2426 of which also showed decreased mRNA levels in the Δ*acsR* mutant. However, in this case log_2_(fold change) was below the threshold of −1.7 (Table S3).

To investigate whether AcsR directly controlled the *clpS*-*ncgl2428-ncgl2427-ncgl2426-acsR* operon, the promoter region of this operon was also studied (Figure S3). In the genome of *C. glutamicum*, *ncgl0635* was predicted to be organized in a putative operon with *ncgl0636*, *ncgl0637*, *ncgl0638*, and *ncgl0639* by RNA-seq-based newtrans analysis (Figure S3). *ncgl0635*, *ncgl0636*, *ncgl0637*, *ncgl0638*, and *ncgl0639* (*irp1*) encoded a putative cytoplasmic siderophore-binding protein, a putative siderophore ABC transporter ATPase, a putative siderophore ABC transporter permease, a putative siderophore ABC transporter permease, and a secreted siderophore-binding lipoprotein Irp1, respectively. The phenomenon indicated that the *irp1*-*ncgl0638*-*ncgl0637*-*ncgl0636*-*ncgl0635* operon encoded SIAS. *ncgl2258* (encoding a putative C4-dicarboxylate binding protein) was organized in a putative operon with *ncgl2256* (encoding a putative C4-dicarboxylate transport system permease), *ncgl2257* (encoding a putative C4-dicarboxylate ABC transporter permease), and *ncgl2259* (encoding a GTP-binding protein LepA) (Figure S1C). Thus, we used *P*_SIAS_ and *P_ncgl2258_* to represent the promoter of the SIAS operon and the *ncgl2259-ncgl2258-ncgl2257-ncgl2256* operon, respectively, in this study.

**Figure 3:**
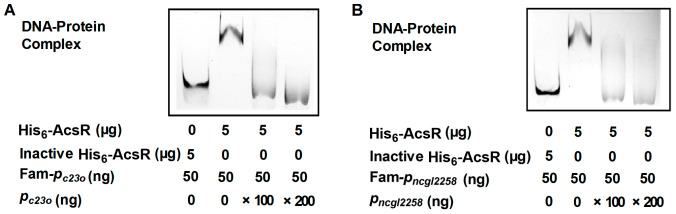
AcsR bound directly to the promoter regions of *c23o* and *ncgl2258* predicted from an in silico search. (**A**,**B**) His_6_-AcsR bound the promoters of *c23o* and *ncgl2258*. Fam 5′-end labeled *c23o* or *ncgl2258* promoter DNA [Fam-*c23o* (**A**) or Fam-*P_ncgl2258_* (**B**)] was incubated with His_6_-AcsR. The protein–DNA complexes were detected by ImageQuant LAS 4000 mini. A 200-fold excess of unlabeled promoter (*P_c23o_* or *P_ncgl2258_*) was used in competition assays for determining the binding specificity of AcsR.

As shown in Figure 3A,B, His_6_-AcsR bound to the promoter regions of *c23o* and *ncgl2258*. The interactions between His_6_-AcsR and Fam-*P_c23o_*, or Fam-*P_ncgl2258_*, were specific, because the formation of His_6_-AcsR-Fam-*P_c23o_* or His_6_-AcsR-Fam-*P_ncgl2258_* complex can be eliminated by adding excessive unlabeled probes (*P_c23o_* or *P_ncgl2258_*) containing *c23o* or *ncgl2258* promoter sequence. However, His_6_-AcsR did not bind to the promoter regions of the SIAS operon, *ncgl0473*, the *clpS*-*ncgl2428-ncgl2427-ncgl2426-acsR* operon, and *genR* (Figure S4A–D). Similarly, no specific shift was observed with DNA fragments covering parts of the coding regions of the *ncgl0635* (+241 to +446 bp), *ncgl0473*, *genR* (+181 to +244 bp), and of the *clpS* (+60 to +326 bp) (Figure S4A–D, Control A); BSA instead of His_6_-AcsR also showed no detectable binding (Figure S4A–D, Control B). According to these results, *c23o* and the *ncgl2259-ncgl2258-ncgl2257-ncgl2256* operon presumably represented direct target genes of AcsR, whereas AcsR regulated its own, the SIAS operon, *ncgl0473*, and *genR* expression indirectly.

### 3.4. Determination of the AcsR Binding Site

**Figure 4:**
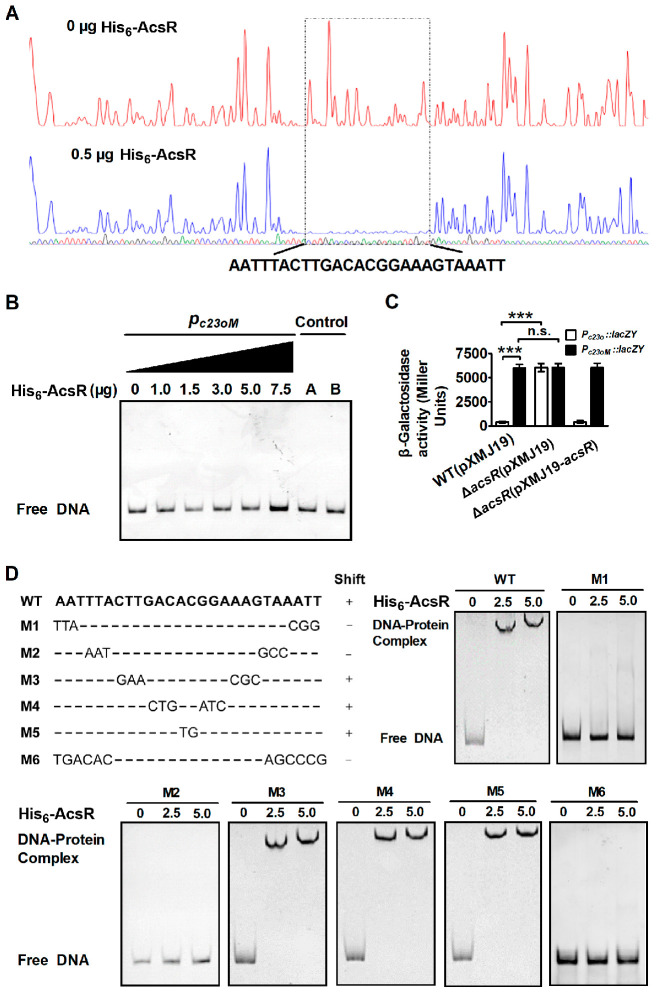
Identification of the AcsR-binding site in the putative *c23o* promoter. (**A**) The binding of AcsR to 26 bp oligonucleotides within the putative *c23o* promoter (*P_c23o_*) was found using the DNase I footprinting assay. (**B**) EMSA was performed to analyze the interaction between His_6_-AcsR and the *c23o* promoter mutating the AcsR protected region (*P_c23oM_*). Fragment amplified from the *c23o* gene coding region using the primers control F1 and control R1 instead of the corresponding promoter (control A) and an irrelevant protein BSA instead of His_6_-AcsR (control B) in the binding assays were used as negative controls. (**C**) A mutation in the AcsR protected region derepressed the *c23o* expression. Relative levels of transcripts were presented as the mean values ± SD calculated from three sets of independent experiments. ***, *p* < 0.001; n.s., not significant. (**D**) Mutational analysis of the 26 bp AcsR protected region in the putative *c23o* promoter. The importance of the protected DNA sequence for AcsR binding was tested in EMSAs with DNA fragments in which two, six or twelve nucleotides of the protected sequence were exchanged, as indicated. “+” indicates that the mutated fragment was bound with the same affinity as the unaltered wild-type (WT) fragment (positive control); “−” indicates that the mutated fragment was not shifted.

DNase I footprinting analysis was performed for locating the precise binding site of AcsR in the promoter region of *c23o* (Figure 4A). A specific 26 bp DNA sequence, AATTTACTTGACACGGAAAGTAAATT, was protected by AcsR (Figure 4A). The protected DNA region extending from −83 to −58 bp upstream of the initiation codon of *c23o* ORF contained a potentially perfect inverted repeat sequence, AATTTACTTN_2_CN_2_GN_2_AAGTAAATT. The sequence was within the proposed −10/35 regions of the promoter, indicating that repression was achieved by inhibiting RNA polymerase binding. To verify the footprinting data, a 180 bp mutation promoter DNA sequence (*P_c23oM_*) was directly synthesized and used for EMSA analysis (Figure S3). Figure 4B showed that *P_c23oM_* eliminated the formation of DNA–protein complexes by EMSA. The promoter DNA mutations in the protected region caused the high β-Galactosidase activities of the *c23o* promoters in the WT(pXMJ19)(*P_c23oM_::lacZY*) and Δ*acsR*(pXMJ19-*acsR*)(*P_c23oM_::lacZY*) strains, similar to those in the Δ*acsR*(pXMJ19)(*P_c23o_::lacZY*) and Δ*acsR*(pXMJ19)(*P_c23oM_::lacZY*) mutants (Figure 4C). Thus, these data confirmed the direct regulatory role of AcsR in the control of *c23o* in *C. glutamicum*.

To determine the importance of the corresponding base pairs within the palindromic sequence, site-directed mutation was introduced into the AcsR-protected region. As shown in Figure 4D, fragments M1–M6 represented derivatives of fragment WT with mutations within the AcsR protected region. In the case of M1 and M2, binding was completely abolished by the mutation, indicating that the six external base pairs [AATTTA-N_14_-TAAATT] were crucial for AcsR binding. An extremely slight decrease in binding was observed for fragment M3, but the binding of fragments M4 and M5 (exchange of the six or two inner bases) was unchanged, suggesting that the accordant exchanged base pairs played only a marginal role in the specific AcsR recognition. To verify the six external base pairs as the AcsR binding site, the 6 bp inverted repeat [AATTTA-N_14_-TAAATT] was present in its altered form (TGACAC-N_14_-AGCCCG, fragments M6). Figure 4D showed that no shift was observed when this sequence was changed. Thus, AcsR specifically binds to the described 6 bp inverted repeat.

A DNA sequence motif similar to the *c23o* promoter (AATTTA-N_14_-TAAATT) was also found in the promoter region of *ncgl2258* (AATTTA-N_13_-TAAATT) and *ncgl2351* (AAATTA-N_9_-TAAATT) (Figure S3). To verify their correlation with AcsR binding, promoter fragments were mutated at the proposed binding sites in the promoter regions of *ncgl2258* (*P_ncgl2258M_*, −270 to −26 bp) and *ncgl2258* (*P_ncgl2351M_*, −146 to −29 bp), which were directly synthesized by PCR, and the mutated sequences are shown in blue below the promoter sequences (Figure S3). *P_ncgl2258M_* and *P_ncgl2351M_* had the same nucleotide sequences as 245 bp *P_ncgl2258_* and 174 bp *P_ncgl2351_* except for mutation sites. EMSA showed that the DNA fragments containing the mutant motifs were not shifted by AcsR (Figure S5). In many cases, the function as transcriptional activator or repressor correlated with a binding site upstream of the −35 region or within or downstream of the −10/35 regions of the promoter, respectively. In Figure S3, the AcsR binding site was located at far upstream of −10/35 regions of the *ncgl2258* promoter, thereby activating transcription, but overlapped with the −10/35 regions of the *ncgl2351* promoter, indicating that repression is achieved by inhibition of RNA polymerase binding or of initiation complex formation. Moreover, RNA-seq data and qRT-PCR analysis revealed that the mRNA levels of *ncgl2258* and *ncgl2351* in the Δ*acsR* mutant were reduced and increased, respectively (Figure 2). According to the results described above, AcsR acted as a transcriptional activator for *ncgl2258* and as a transcriptional repressor for *ncgl2351*.

In addition, the putative promoter regions of the genes encoding homologous C23Os in *C. crudilactis, C. deserti*, and *C. callunae* shared a similar palindromic sequence (Figure S6). The identification of the AcsR binding sites in the promoter regions of *c23o*, *ncgl2258*, and *ncgl2351* allowed us to deduce a possible binding motif of AcsR (AATTTA-N_9_–_14_-TAAATT) (Figure S6). The size of the binding motif was consistent with AcsR binding as a homodimer at the site, a conclusion supported by size-exclusion chromatography evidence for uncomplexed AcsR existing as a dimer (Figure S7). However, the sequence of this site was not similar to binding sites from *Rhodopseudomonas palustris* MarR-type BadR (ATTTGAT-N_4_-ATCAAAT) [43] and *Bifidobacterium longum* MarR-type BmrR (ATTGTTG-N_6_-CAACAAT) [44].

### 3.5. AcsR Was Required for Catechol-Dependent Regulation of C23O

In bacteria, phenolic compounds are typically metabolized via the catechol pathway, in which catechol undergoes ring-opening reactions catalyzed by either catechol 1,2-dioxygenase (C12O, ortho cleavage) or catechol 2,3-dioxygenase (C23O, meta cleavage), ultimately integrating into central carbon metabolism [45]. The function of C12O (encoded by *ncgl2319*) has been validated, whereas the putative C23O has not yet been characterized. To verify the function and expression of the potential C23O from *C. glutamicum* under the induction of aromatic compounds, its biochemical activity and transcription were assayed. As shown in Table S4, the putative C23O from *C. glutamicum* exhibited substantial ring-cleavage extradiol dioxygenase activity towards catechol, comparable to that of C23O from the Gram-positive *Planococcus* sp. Strain S5 [40], thereby confirming its identity as a 2,3-dioxygenase.

**Figure 5:**
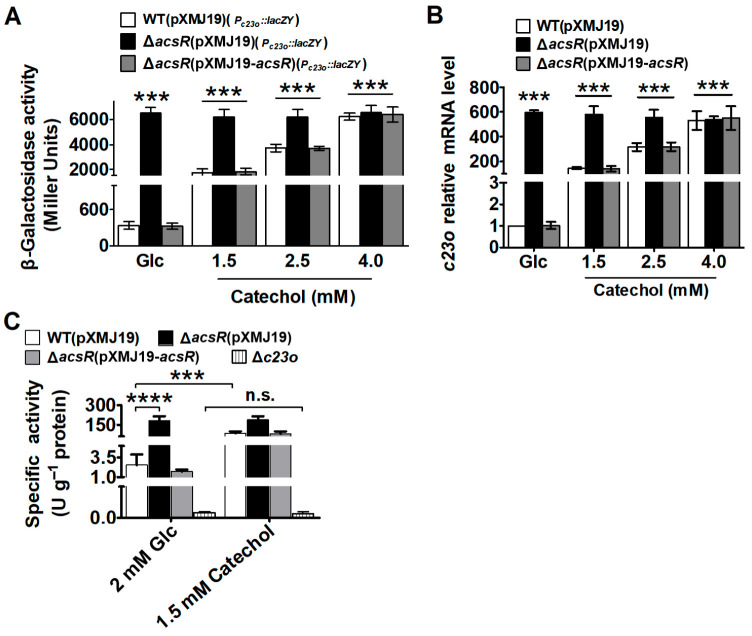
AcsR negatively regulated *c23o* expression. (**A**) β-Galactosidase activities of the *c23o* promoter in WT(pXMJ19), Δ*acsR*(pXMJ19) mutant, and complementary Δ*acsR*(pXMJ19-*acsR*). Significant differences were analyzed by comparing the promoter activities between the corresponding strains grown on CGXII MM with catechol and WT(pXMJ19) strains grown on CGXII MM with 2 mM Glc. *** *p* < 0.001. (**B**) qRT-PCR analyses of the *c23o* expression in WT(pXMJ19), Δ*acsR*(pXMJ19) mutant, and complementary Δ*acsR*(pXMJ19-*acsR*) strains. The mRNA levels were presented relative to the value obtained from WT(pXMJ19) cells grown on CGXII MM with 2 mM Glc. Relative transcript level of WT(pXMJ19) strains grown on CGXII MM with 2 mM Glc was set at a value of 1.0. Significant differences were analyzed by comparing the mRNA levels between the corresponding strains grown on CGXII MM with catechol and WT(pXMJ19) strains grown on CGXII MM with Glc. *** *p* < 0.001. (**C**) In vivo expression of specific activity from *C. glutamicum* C23O proteins. The data shown were the average of three independent experiments. The error bars indicated standard deviations of the mean. *** *p* < 0.001; **** *p* < 0.0001; n.s., not significant.

Then, we used catechol as an effector molecule to assess its effect on AcsR–DNA complex formation. A clear backshift was observed for catechol but not for 10 mM Glc (Figure S8). Consistently, the derepression of C23O transcription by catechol was mediated via AcsR in vivo. As shown in Figure 5A, regardless of Glc or catechol as an inducer, the *c23o* promoter activities in the Δ*acsR*(pXMJ19)(*P_c23o_::lacZY*) strain were significantly higher than those in the WT(pXMJ19)(*P_c23o_::lacZY*) and Δ*acsR*(pXMJ19-*acsR*)(*P_c23o_::lacZY*) strains grown with Glc, indicating that AcsR indeed negatively regulated the expression of *c23o*. However, the *c23o* promoter showed obviously higher LacZY activities in WT(pXMJ19)(*P_c23o_::lacZY*) and Δ*acsR*(pXMJ19-*acsR*)(*P_c23o_::lacZY*) strains induced with catechol than with Glc. In addition, LacZY activity analysis showed that the response of WT(pXMJ19)(*P_c23o_::lacZY*) and Δ*acsR*(pXMJ19-*acsR*)(*P_c23o_::lacZY*) strains to catechol was expressed in a dose-dependent manner (Figure 5A). Through qRT-PCR analysis, a similar regulation mode of *c23o* by AcsR was also observed at the mRNA transcriptional level (Figure 5B). These findings demonstrated that the transcription level of *c23o* increased in response to catechol. To further confirm catechol as an inducer, the *c23o* expression was also investigated in WT, Δ*c23o* mutant, and Δ*acsR* mutant exposed to catechol via the biochemical activity of C23O proteins. As shown in Figure 5C, the Δ*c23o* mutant had low aromatic ring-fission activity against catechol in cell extracts, exhibiting approx. one in twenty-six of the enzyme activity measured in extracts from WT cells. The relative enzyme activity of the Δ*acsR* mutant was determined to be about 69-fold higher than in extracts from WT cells. When strains were exposed to catechol, significantly increased activities in WT were observed compared to the corresponding strain without catechol conditions (Figure 5C). This was consistent with the levels of catechol-induced mRNA expression, as shown above. These results indicated that catechol served as an inducer of the expression of *c23o*. Thus, the major function of AcsR was revealed to be the inactivation of C23O in the absence of external catechol. The addition of catechol induced the dissociation of AcsR from *P_c23o_*. All of these results clearly demonstrated that *c23o* was upregulated with the increase in catechol concentration, indicating that catechol inhibited the binding of AcsR to DNA and induced the expression of the *c23o* gene.

## 4. Discussion

The *ncgl2259*-*ncgl2258*-*ncgl2257*-*ncgl2256* operon codes for the C4-dicarboxylate ABC transport system, which participates in the translocation of C4-dicarboxylates across biological membranes. C4-dicarboxylates serve as key metabolic intermediates and play a central role in cellular physiology. Under aerobic conditions, they participate in the tricarboxylic acid (TCA) cycle, whereas in anaerobic bacteria, they contribute to energy-conserving fermentation and respiratory processes. Additionally, *ncgl2248*, which encodes isocitrate lyase, plays a critical role in metabolic adaptation to environmental changes. Isocitrate lyase catalyzes the reversible cleavage of isocitrate into succinate and glyoxylate—a pivotal step in the glyoxylate cycle, which functions as an anaplerotic pathway to replenish TCA cycle intermediates during growth on fatty acid substrates [46]. Bacteria fought adversity by improving energy production [47]. Given the downregulation of *ncgl2258* and *ncgl2248* expression in the Δ*acsR* mutant, we hypothesize that the phenomenon that the Δ*acsR* mutant showed sensitivity to antibiotics, oxidants, and heavy metals compared to WT was related to reduced TCA cycle activity and diminished energy production.

*C. glutamicum* efficiently degrades aromatic hydrocarbons and utilizes aromatic compounds as primary carbon and energy sources for growth [45]. Typically, aerobic biodegradation of aromatics involves their initial conversion into dihydroxylated intermediates—such as catechol or its alkyl- or chlorosubstituted derivatives—which are subsequently metabolized via intradiol or extradiol dioxygenases [45]. Six aromatic compound degradation-related genes (*ncgl0971*, *c23o*, *ncgl1212*, *benA* (*ncgl2320*), *benB* (*ncgl2321*), and *genR*) were increased in abundance by at least 1.8-fold in the Δ*acsR* mutant. Among them, *c23o* and *genR* were strongly upregulated. C23O, an extradiol dioxygenase, cleaves the aromatic ring at the meta position of dihydroxylated substrates and catalyzes the conversion of catechol to 2-hydroxymuconic semialdehyde, which subsequently enters the TCA cycle [40]. Catechol was generated from many aromatic compounds, such as benzyl alcohol and benzoate, via BenABCD (NCgl2320/NCgl2321/NCgl2322/NCgl2323) [45]. The IclR-type regulator GenR was shown to trigger the expression of gentisate 1,2-dioxygenase (NCgl2920), maleylpyruvate isomerase (NCgl2918), and fumarylpyruvate hydrolase (NCgl2919), which were involved in GA/3-HBA catabolism [3]. Deletion of *genR* abolishes the ability of *C. glutamicum* to grow on GA and 3-HBA. Notably, GA and substituted GA derivatives represent key intermediates in the aerobic degradation of numerous aromatic compounds, including 3-HBA [3]. Survival experiments exhibited that the *C. glutamicum* Δ*acsR* mutant had decreased sensitivity to aromatic compounds. Moreover, *c23o* was upregulated in response to increasing catechol concentration. Therefore, we speculated that the increased resistance of the Δ*acsR* mutant to aromatic compounds was related to upregulated expression of *ncgl0971*, *c23o*, *ncgl1212*, *benA*, *benB*, and *genR*.

Our RNA-seq-based results displayed that AcsR positively controlled the expression of *ncgl0734* (*whcE*), *ncgl0401* (*mrx3*), and *ncgl2286* (*ahpD*). The transcriptional regulator WhcE played roles in oxidative stress responses [48]. The *whcE* mutant exhibited reduced survival under heat stress and increased susceptibility to thiol-specific oxidants, such as diamide, as well as redox-cycling compounds, such as MEN and plumbagin. Thioredoxin reductase (encoded by *trxB*), which was also involved in various oxidative and redox stresses, was under the control of WhcE [48]. Mrx3 contributes to protection against oxidative stress by functioning as a disulfide oxidoreductase with Trx-like activity [49]. AhpD, encoding an alkyl hydroperoxidase reductase, played a role in defense against peroxides such as H_2_O_2_ and related compounds [50]. Mols found that heavy metals, antibiotics, and alkylating agents could induce the generation of ROS [8]. Thus, we speculated that AcsR exerts its influence by regulating the expression of genes related to redox processes. In addition, the deletion of *acsR* increased heavy metal sensitivity and the expression of the heavy metal (nickel) transport system permease gene *ncgl2351*, suggesting that many heavy metal ions entered the cell and caused damage when *acsR* was deleted.

As a member of the MarR family of transcriptional regulators, AcsR belongs to a class of proteins that control a variety of microbial functions, including aromatic compound degradation, substrate catabolism, and stress responses (Figure 2). On a functional level, AcsR exhibited some similarities to *R. palustris* BadR [42], *E. coli* EmrR [51], and *Deinococcus radiodurans* HucR [52], which formed one protein–DNA complex when they were combined with DNA containing the appropriate cognate promoter. Furthermore, the binding site of AcsR contained an inverted repeat structure that was moderately similar to the inverted repeat detected in the binding site of BadR. In addition, the binding sites of AcsR, BadR [46], EmrR [51], and HucR [52] also differed in sequence, size, reverse repeat spacing, and position relative to the transcription start site and start codons of the target genes. While most MarR-family regulators act as repressors, certain members, such as BadR and SlyA, function as activators of their respective operons [43,53]. The genomic organization of MarR-encoding genes relative to their target genes varies considerably: some are divergently transcribed (e.g., *mexR*), others are co-transcribed within the same operon (e.g., *emrR*) [51], and still others reside in separate operons (e.g., *badR*) [43]. Despite this functional diversity, MarR-like proteins share conserved characteristics, including responsiveness to antibiotics and adverse stress [54] and a central conserved motif implicated in DNA binding [55].

Comparative transcriptomic analysis of the WT strain and the Δ*acsR* mutant, combined with DNA-binding assays using purified AcsR protein, enabled the identification of direct target genes and the deduction of a consensus DNA-binding motif. A perfect 6 bp inverted repeat with the consensus sequence (5′-AATTTA-N_9-14_-TAAATT-3′) was identified in the upstream regions of *c23o*, *ncgl2258*, and *ncgl2351* genes. EMSAs demonstrated specific binding of dimeric AcsR to this motif, and catechol blocked binding. Catechol induced the expression of *c23o* in vivo.

Homologs of AcsR, C23O, NCgl2258, and similar genetic organizations were found in other members of the *Corynebacterium* species (Figure S1B–D), suggesting that they shared a similar regulatory mechanism or function. The present work may open new possible avenues for regulating the aromatic compound degradation and iron acquisition.

## 5. Conclusions

In this study, we characterize a novel MarR-type regulator, AcsR, encoded by *ncgl2425* in *C. glutamicum*. Through comparative transcriptome analysis using RNA-seq of Δ*acsR* and WT, combined with EMSAs and DNase I footprinting assays using purified AcsR protein, we identified direct target genes regulated by AcsR. Specifically, the expression of *ncgl2258*, encoding an ABC-type C4-dicarboxylate-binding periplasmic protein, was activated by AcsR. In contrast, *ncgl2007*, encoding C23O, was repressed by AcsR. Additionally, *ncgl0635,* organized in an operon with *ncgl0639*-*ncgl0638*-*ncgl0637*-*ncgl0636* and encoding components of an ABC-type SIAS, was indirectly regulated by AcsR. Dimeric AcsR binds specifically to a perfect 6 bp inverted repeat sequence located within the promoter regions of its target genes. Catechol was identified as a ligand effector of AcsR, triggering dissociation of the AcsR–DNA complex in vitro. Notably, in vivo experiments showed that the addition of an iron chelator induced expression of the SIAS operon, whereas catechol supplementation induced *c23o* expression. Furthermore, the Δ*acsR* mutant also exhibited enhanced resistance to various aromatic compounds but heightened sensitivity to oxidants, antibiotics, Cu^2+^, Cr^6+^, and Cd^2+^. Collectively, these findings demonstrate that AcsR plays a pivotal role in stress resistance in *C. glutamicum*, significantly expand our understanding of AcsR functionality, and provide new insights into the coordinated regulation of aromatic compound degradation, iron homeostasis, and stress response.

## Data Availability

The data presented in this study are openly available in Transcriptome data of *Corynebacterium glutamicum* at https://www.ncbi.nlm.nih.gov/bioproject/?term=PRJNA1058395, reference number PRJNA1058395.

## Supplementary Materials

The following supporting information can be downloaded at: https://www.mdpi.com/article/10.3390/microorganisms14040920/s1, Table S1. Bacterial strains and plasmids used in this study; Table S2. Primers used in this study; Table S3. Genome-wide comparison of mRNA levels in *C. glutamicum* acsR mutant (Δ*acsR*) and *C. glutamicum* RES167 parental strain (WT) using RNA-seq analysis; Table S4. Kinetic parameters of *C. glutamicum* C23O protein using catechol as the substrate. Figure S1. Comparison of the gene organization and genome locus in *C. glutamicum* and related species; Figure S2. Cell morphology of *C. glutamicum* WT and Δ*acsR*; Figure S3. The promoter regions of the AcsR-regulated genes; Figure S4. Electrophoretic mobility shift assays (EMSAs) were performed to analyze the interactions between His_6_-AcsR and DNA fragments covering the putative promoter regions of *ncgl0473* (A), the SIAS operon (an ABC-type siderophore-mediated iron acquisition system) (B), *genR* (C), or the *clpS*-*ncgl2428*-*ncgl2427*-*ncgl2426*-*acsR* operon (D); Figure S5. The promoter DNA mutations in the predicted AcsR-binding sites prevented the binding of AcsR; Figure S6. Sequence of the promoter region of *C. glutamicum c23o* aligned to putative promoter regions from other *Corynebacterium* species; Figure S7. Purification of His_6_-AcsR and determination of the native molecular mass by gel filtration; Figure S8. Inhibition of the DNA binding of AcsR by catechol. References [3,56,57,58] are cited in the supplementary materials.
