# Supplementary material for: Functional Identification of AcsR, a MarR Family Transcriptional Regulator Involved in the Regulation of Aromatic Compound-Degrading Genes in Corynebacterium glutamicum
Source: Microorganisms. 2026 Apr 18;14(4):920. doi: 10.3390/microorganisms14040920 (PMC13118786; doi:10.3390/microorganisms14040920)
Supplement: Supplementary file 1 [file microorganisms-14-00920-s001.zip › microorganisms-4233791-supplementary.pdf]

Supplemental Data

Table S1. Bacterial strains and plasmids used in this study.

Strains or plasmids	Relevant genotype description	References
**Strains**		
Corynebacterium glutamicum		
RES167	Restriction-deficient mutant of ATCC13032, Δ(*cglIM-cglIR-cglIIR*)	[1] University of Bielefeld
Δ*acsR*	*acsR* deleted in RES167	This study
Δ*c23o*	*c23o* deleted in RES167	This study
WT(pXMJ19)	RES167 parental strain containing pXMJ19	[2]
Δ*acsR*(pXMJ19)	Δ*acsR* containing pXMJ19	This study
Δ*acsR*(pXMJ19-*acsR*)	Δ*acsR* containing pXMJ19-*acsR*	This study
WT(pXMJ19)(P_{c23o}*::lacZY*)	WT(pXMJ19) containing P_{c23o}*::lacZY*	This study
Δ*acsR*(pXMJ19)(P_{c23o}*::lacZY*)	Δ*acsR* containing P_{c23O}*::lacZY*	This study
E. coli		
BL21(DE3)	Host for expression vector pET28a	Novagen (catalog no. 69387–3)
JM109	*recA*1 supE44 endA1 hsdR17 gyrA96 relA1 thi Δ(*lac-proAB*)F′(*traD36 proABlacI^q lacΔZM15*)	Stratagene (catalog no. 200235)
**Plasmids**		
pK18mob*sacB*	Suicide plasmid carrying *sacB* for selecting double crossover in *C. glutamicum*, Km^r	[3]
pK18mob*sacB*-Δ*acsR*	Construct used for in-frame deletion of *acsR*	This study
pK18mob*sacB*-Δ*c23o*	Construct used for in-frame deletion of *c23o*	This study
pK18mob*sacB*-P_{c23o}*::lacZY*	P_{c23o}*::lacZY* fusion in pK18mob*sacB*	This study
pK18mob*sacB*-P_{c23oM}*::lacZY*	P_{c23oM}*::lacZY* fusion in pK18mob*sacB*	This study
pXMJ19	Shuttle vector (Ptac lacI^q pBL1 oriV$_{C.\ glutamicum}$ pK18	[4]
pXMJ19-*acsR*	*acsR* cloned into pXMJ19 for complementation	This study
pET28a	Expression vector with N-terminal hexahistidine	Novagen
pET28a-*acsR*	*acsR* in pET28a	This study
pET28a-*c23o*	*c23o* in pET28a	This study

References

1. Schäfer A, Tauch A, Jager W, Kalinowshi J, Thierbach G, Pühler A.Small mobilizable multi-purpose cloning vectors derived from the *Escherichia coli* plasmids pK18 and pK19: selection of defined deletions in the chromosome of *Corynebacterium glutamicum*. Gene.1994; 145: 69-73.

2. Jakoby M, Ngouoto-Nkili CE, Burkovski A. Construction and application of new *Corynebacterium glutamicum* vectors. Biotechnol Tech. 1999; 13: 437-441.

3. Karimova G, Pidoux J, Ullmann A, Ladant, D. A bacterial two-hybrid system based on a reconstituted signal transduction pathway. Proc Natl Acad Sci USA.1998; 95: 5752-5756.

4. Shen XH, Huang Y, Liu SJ. Genomic analysis and identification of catabolic pathways for aromatic compounds in *Corynebacterium glutamicum*. Microbes Environ. 2005; 20:160-167.

Table S2. Primers used in this study.

Primers	5'-3' sequence	
acsRcom-F	CCCAAGCTTCGACTCTAGAGGATCCAAAGGAGGACAACCATGACAAGTGAGAATTCCGAATCC (*Hin*dIII)	For cloning *acsR* into pXMJ19
*acsR*com-R	ACGCGTCGACCTACACCCGGGGCAGCCTGTTGGAC (*Sal*I)	
O_{acsR}-F	ATGGGTCGCGGATCCGAATTCATGACAAGTGAGAATTCCGAATCC (*Eco*RI)	For cloning *acsR* into pET28a
O_{acsR}-R	GTGGTGGTGGTGGTGCTCGAGCTACACCCGGGGCAGCCT (*Xho*I)	
O_{c23o}-F	CCGGAATTCATGGCACTTCTTGAGCAGAGCATC (*Sal*I)	For cloning *c23o* into pET28a
O_{c23o}-R	GGACTCGAGTTATCGGTTCAGAAGTTCATCTG (*Xho*I)	
D_{acsR}-F1	CTATGACATGATTACGAATTCCAACACGAATTTCAGCACCACT (*Eco*RI)	To generate pK18*mobsacB*-Δ*acsR*
D_{acsR}-R1	CAATCTTCTTCGCTCATCGGTTAGCCAAATGTC	
D_{acsR}-F2	CCGATGAGCGAAGAAGATTGTGAACCGACTCG	
D_{acsR}-R2	CAGGTCGACTCTAGAGGATCCATTCCAATCGGCGCATCA (*Bam*HI)	
D_{c23o}-F1	CGCGGATCCATTGGTTGCGTTCACCACCTCTGG (*Bam*HI)	To generate pK18*mobsacB*-Δ*c23o*
D_{c23o}-R1	TTGAAGAGTAGTAACTAGTCATACC	
D_{c23o}-F2	GGTATGACTAGTTACTACTCTTCAAAGGATCTGGACATGCTGGTCTCACG	
D_{c23o}-R2	CCCAAGCTTCACCGATCAGTGGCACACCCAAGTC (*Hin*dIII)	
P_{c23o}-F1	TCCCCCGGGGAAAAGCCAGAGGCTGTCACCTCC (*Sma*I)	To generate pK18*mobsacB*-P_{c23o}::*lacZY*
P_{c23o}-R1	CTCAAGAAGTGCCATGGTGTTTCATC	
lacZY-F1	GATGAAACACCATGGCACTTCTTGAGACTAGTACTAGTATGACCATGATTACGGATTC (*Spe*I)	
lacZY-R	AAAACTGCAGTTAAGCGACTTCATTCACCTG (*Pst*I)	
P_{c23o}-mutation-F	GCTGCCATTGACACTGTTCACGCGAAGGTGAACAATGGTGTTCAACAG	Site directed mutation in the P_{c23o} region
P_{c23o}-mutation-R	TTCGCGTGAACAGTGTCAATGGCAGCCTTAAATGAGGCGGATTTCCCC	
Qc23o-F	CAAGAATCACGCTCACGTTTTGTG	RT-PCR
Qc23o-R	GTACTGGTTTGGATCCAAGTAGATG	
Qncgl0037-F	GGTGGGCGAGATATTTCCAGCATG	RT-PCR
Qncgl0037-R	GCGCGCCACAATGGCTTCATCGTC	
Qncgl0064-F	CTGCAGGAAGAGGGCCACAATGTG	RT-PCR
Qncgl0064-R	GGTGTCGTAGAGCTCGCTGAGCTG	
Qncgl0473-F	CATCGCAGTTGGTGCCATCTTG	RT-PCR

Qncgl0473-R	GAGTGGGCTTTTGTATTTCCTAC	
Qncgl0970-F	CACCAGGCTTATTCACTTACGACG	RT-PCR
Qncgl0970-R	CCGTCCATTCTGGGGCTGCGGGAG	
Qncgl1404-F	CGGTCTTGTCCGCGCTACGGAAGG	RT-PCR
Qncgl1404-R	GTGCGCGAAGCGGCCCACCAAAAC	
Qncgl2258-F	CCCAAGGCACCCGAAATCTGTAC	RT-PCR
Qncgl2258-R	CGCGGTATATACCTCGCCGTAAG	
Qncgl2351-F	CTGCCAGGCGGCGGTGCAGCTCAC	RT-PCR
Qncgl2351-R	GAGTACACGAACAGTGCCGCAACG	
Qncgl2975-F	CTATCAAGAACTACACCGTCGAAG	RT-PCR
Qncgl2975-R	GCAGCCTTGACAGCCTCGTCGGTG	
16S rRNA-F	ACCTGGAGAAGAAGCACCG	RT-PCR
16S rRNA-R	TCAAGTTATGCCCGTATCG	
EclpS-F	GGTTTCCATTCGGTGGTCCTTCCGCC	To produce 266-bp EMSA *clpS* promoter DNA
EclpS-R	GACAAATAGCCTAGTCAACAACTATGCG	
EgenR-F	ATTGCAAAGCTCTAATCGCTTAAATC	To produce 64-bp EMSA *genR* promoter DNA
EgenR-R	CATGGTGTTTCATCCTTTAAGTGTTG	
Encgl0473-F	GCAAGGAAACCTTAAATCCAGC	To produce 174-bp EMSA *ncgl0473* promoter DNA
Encgl0473-R	AGTTATAAAACTAGGTCTGATC	
Encgl2258-5´Fam-F	ATGTGTTCTCACCTTAAATATCGGC	To produce 245-bp EMSA *c23o* promoter DNA
Encgl2258-R	ATAACGGTGTCCTACTCTACCTGAG	
Encgl2258-F	ATGTGTTCTCACCTTAAATATCGGC	unlabeled competitor DNA
Encgl2258-R	ATAACGGTGTCCTACTCTACCTGAG	
Encgl2351-F	GGCTACCTGCTTATCGGTTGTG	To produce 174-bp EMSA *ncgl2351* promoter DNA
Encgl2351-R	CTAGGCTTACCTCCAGTGAATC	
Ec23o-5´Fam-F	CTCTAATCGCTTAAATCTTTC	To produce 180-bp EMSA *c23o* promoter DNA
Ec23o-R	GGTGTTTCATCCTTTAAGTGTTG	
Ec23o-F	CTCTAATCGCTTAAATCTTTC	unlabeled competitor DNA
Ec23o-R	GGTGTTTCATCCTTTAAGTGTTG	
P_{c23o}-footprinting-F	CTCTAATCGCTTAAATCTTTC	Footprinting
P_{c23o}-footprinting-R	GGTGTTTCATCCTTTAAGTGTTG	
$P_{ncgl0473}$-footprinting-F	GCAAGGAAACCTTAAATCCAGC	Footprinting

**$P_{ncgl0473}$-footprinting-R**	**AGTTATAAAACTAGGTCTGATC**	
**Control-F1**	**CACACCGCTTTCCTCTTCGATACC**	**To produce 180-bp control EMSA *c23o* promoter DNA**
**Control-R1**	**CCATGCACTTCGAGGGCGGTCAAC**	
**Control-F2**	**GCATTGGGTGTGACTCCTGTCGGC**	**To produce 206-bp control EMSA SIAS promoter DNA**
**Control-R2**	**TGGGTGATGCCAGAGTAGGACGCC**	
**Control-F3**	**GTAGCGAGCAACCTCGAAGTGG**	**To produce 245-bp control EMSA *ncgl2258* promoter DNA**
**Control-R3**	**CCATGCACTTCGAGGGCGGTCAAC**	
**Control-F4**	**GCAGTGGAAAGTGTGGAATCAAG**	**To produce 266-bp control EMSA *clpS* promoter DNA**
**Control-R4**	**TTGTCCTTCTCGCCAGAACTCACC**	
**Control-F5**	**CGGGGTTTTGCAGTCAGAAGCGAG**	**To produce 64-bp control EMSA *genR* promoter DNA**
**Control-R5**	**CGGAGGTCGCCAATGCAGAGCCTG**	
**Control-F6**	**TATCTATTGGCATCGCAGTTGGTG**	**To produce 174-bp control EMSA *ncgl0473* promoter DNA**
**Control-R6**	**ATCCATACCCTGCCATCTTAGAG**	
**Control-F7**	**GGCAGTCCTGTCCGGCTGCACAGC**	**To produce 174-bp control EMSA *ncgl2351* promoter DNA**
**Control-R7**	**CAAGGTTTCAGGATCCTGGTAGAG**	

Underlined sites indicate restriction enzyme cutting sites added for cloning. Letters in italic denote the mutation sites in overlap PCR for site-directed mutation.

Table S3 Genome-wide comparison of mRNA levels in *C. glutamicum acsR* mutant (Δ***acsR***) and *C. glutamicum* RES167 parental strain (WT) using RNA-seq analysis. **The mRNA ratio represented mean values from three independent microarray experiments starting from independent cultures. The strains were cultivated in LB medium, and mRNA was isolated in the exponential growth phase. [a] Log_2^{Ratio} was defined by log_2(the gene mRNA expression ratio of the *Corynebacterium glutamicum* Δ*acsR* mutant to *Corynebacterium glutamicum* RES167 parental strain (WT)). Log_2^{Ratio} values of higher than +1.7 or lower than -1.7 (corresponding to mRNA ratio Δ*acsR*/WT of >3.25 and < 0.308, respectively) were considered to be significant. The table included those genes that showed a ≥3.25-fold changed mRNA level (increased or decreased) in at least two of the three experiments and that had a *p* value < 0.05[b]. The genes were ordered according to their position on the genome. The mRNA ratios for the genes *ncgl0379*, *ncgl2322*, *ncgl2323*, *ncgl2426* and *ncgl2483* were marked with an asterisk, as their mRNA ratios were not within the defined range. However, the genes were included, as they were part of operons of which the other genes fulfilled the selected criteria.**

Accession no.	Gene name	Predicted function	Log_2 Ratio [a]	*p*-value [b]
**Genes with an enhanced mRNA level in Δ*acsR* mutant**				
NCgl0037		Iron complex transport system ATP-binding	3.62	7.61E-05
NCgl0357		Putative regulatory protein	9.58	0.014
NCgl0375		Cation transporting ATPase	2.89	8.61E-03
NCgl0376		Cell-surface hemin receptor	2.91	1.1E-03
NCgl0377		Putative cell-surface hemin receptor	6.68	0.01
NCgl0379*		Heme transport system ATP-binding protein	1.68	6.76E-07
NCgl0473		Putative membrane protein	10.98	0.015
NCgl0482		Iron complex transport system ATP-binding	2.18	8.76E-04
NCgl0580		Permeases of the drug/metabolite transporter	2.57	1.27E-05
NCgl0635	*sip*	Siderophore-interacting protein	9.58	0.031
NCgl0638		ABC transporter permease	4.27	3.47E-04
NCgl0777		Iron-siderophore transport system permease	4.28	3.45E-04
NCgl0960		Allophanate hydrolase subunit 2	2.84	1.12E-09
NCgl0961		Allophanate hydrolase subunit 1	2.64	4.26E-08
NCgl0962		LamB/YcsF family protein	2.36	9.08E-06
NCgl0963		Putative Mn^{2+}/Fe^{2+} transporter	1.96	1.16E-04
NCgl0964		Putative membrane protein	1.71	6.32E-04
NCgl0970		Putative protein	10.44	0.023
NCgl0971		NADPH-dependent FMN reductase	2.45	3.93E-11
NCgl1123		Polyisoprenoid-binding protein	1.97	0.017
NCgl1209		ABC-type Fe^{3+}-siderophore transport system	2.08	0.015
NCgl1212		Predicted dinucleotide-binding enzyme	9.80	0.019
NCgl1514		Glucose-6-phosphate 1-dehydrogenase	1.84	1.02E-07
NCgl1462		Putative protein	9.44	0.033
NCgl1651		Putative secreted protein	9.60	0.022
NCgl1740		Putative methyltransferase	1.85	0.0013
NCgl2006		Glucan phosphorylase	9.63	0.027
NCgl2007	*c23o*	Catechol 2, 3-dioxygenase	9.29	0.025

NCgl2028		Hydroxypyruvate isomerase	1.83	3.37E-06
NCgl2320	*benA*	Benzoate 1,2-dioxygenase small subunit	1.99	1.98E-05
NCgl2321	*benB*	Benzoate 1,2-dioxygenase large subunit	1.87	4.49E-06
NCgl2322*	*benC*	Benzoate 1,2-dioxygenase ferredoxin reductase	1.67	3.49E-06
NCgl2323*	*benD*	Cis-diol dehydrogenase	1.65	1.79E-04
NCgl2351		Heavy metal (nickel) transport system permease	2.13	5.66E-08
NCgl2367		Alkaline phosphatase family protein	2.73	0.0081
NCgl2683		Sodium: glutamate symporter	2.14	1.98E-07
NCgl2684	*arsR*	Predicted ArsR family transcriptional regulator	3.77	0.03
NCgl2685		Predicted Co^{2+}/Zn^{2+}/Cd^{2+} cation transporter	2.77	2.21E-04
NCgl2714		Sirohydrochlorin chelatase	1.78	5.36E-05
NCgl2696		Putative protein	2.51	0.0054
NCgl2858		Putative protein	1.79	1.27E-07
NCgl2861		Fibronectin type III domain containing protein	2.55	0.0018
NCgl2913	*msr*	Malonic semialdehyde reductase	6.32	0.014
NCgl2921	*iclR*	IclR family transcriptional regulator	11.6	1.01E-10
NCgl2961		Proline-betaine transporter	1.96	4.26E-04
NCgl2975		Putative copper chaperone	7.53	0.0084
Genes with a decreased mRNA level in Δ*acsR* mutant				
NCgl0064		ATPase related to phosphate starvation-inducible	-3.56	0.009
NCgl0122		3-oxoacyl-acyl-carrier-protein synthase III	-1.94	4.03E-04
NCgl0401	*mrx3*	Mycoredoxin 3	-1.75	3.34E-07
NCgl0412		Putative iron ABC transporter	-1.81	1.47E-06
NCgl0699		Putative protein	-2.94	8.14E-03
NCgl0465		Putative cation exporting P-type ATPase A	-1.80	1.28E-07
NCgl0734	*whcE*	WhiB family transcriptional regulator, redox-	-3.85	0.014
NCgl1025		ADP-ribose pyrophosphatase	-3.02	5.72E-06
NCgl1213		L-glyceraldehyde 3-phosphate reductase	-2.55	3.45E-05
NCgl1403		Phosphonate ABC transporter permease	-3.12	1.90E-06
NCgl1404	*phnC*	Phosphonate import ATP-binding protein PhnC	-3.00	4.42E-05
NCgl1405		Phosphate/phosphite/phosphonate ABC	-3.11	6.15E-07
NCgl1406		Putative phosphoglycolate phosphatase	-1.72	0.032
NCgl1427		Cobalamin biosynthesis protein	-2.17	4.03E-03
NCgl1488		Putative cation exporting P-type ATPase	-1.73	2.855E-05
NCgl1867		Putative protein	-2.00	0.012
NCgl2065		The drug/metabolite transporter	-1.77	1.01E-04
NCgl2130		Succinate exporter	-1.80	6.05E-06
NCgl2248		Isocitrate lyase	-1.77	0.027
NCgl2258		Putative C4-dicarboxylate binding protein	-9.10	0.03
NCgl2286	*ahpD*	Alkylhydroperoxidase	-3.94	0.01
NCgl2399		Gluconate kinase	-1.77	3.91E-04

**NCgl2426***		**Rhomboid family intramembrane serine protease**	**-1.78**	**5.90E-08**
**NCgl2429**	***clpS***	**ATP-dependent Clp protease adaptor protein**	**-7.30**	**8.27E-08**
**NCgl2483***		**Phosphate ABC transporter ATP-binding protein**	**-1.77**	**9.69E-07**
**NCgl2484**	***pstA***	**Phosphate ABC transporter, permease protein**	**-4.62**	**3.93E-08**
**NCgl2485**	***pstC***	**phosphate ABC transporter, permease protein**	**-3.76**	**0.014**
**NCgl2859**		**Putative copper-exporting P-type ATPase**	**-1.83**	**1.25E-03**

Table S4. Kinetic parameters of *C. glutamicum* C23O protein using catechol as the substrate.

**Enzyme**	K_m **(μM)**	k_{cat} **(s^{-1})**	k_{cat} / K_m **(μM^{-1} s^{-1})**
**C23O**	**5.97±3.6**	**2.64±13.7**	**0.44**

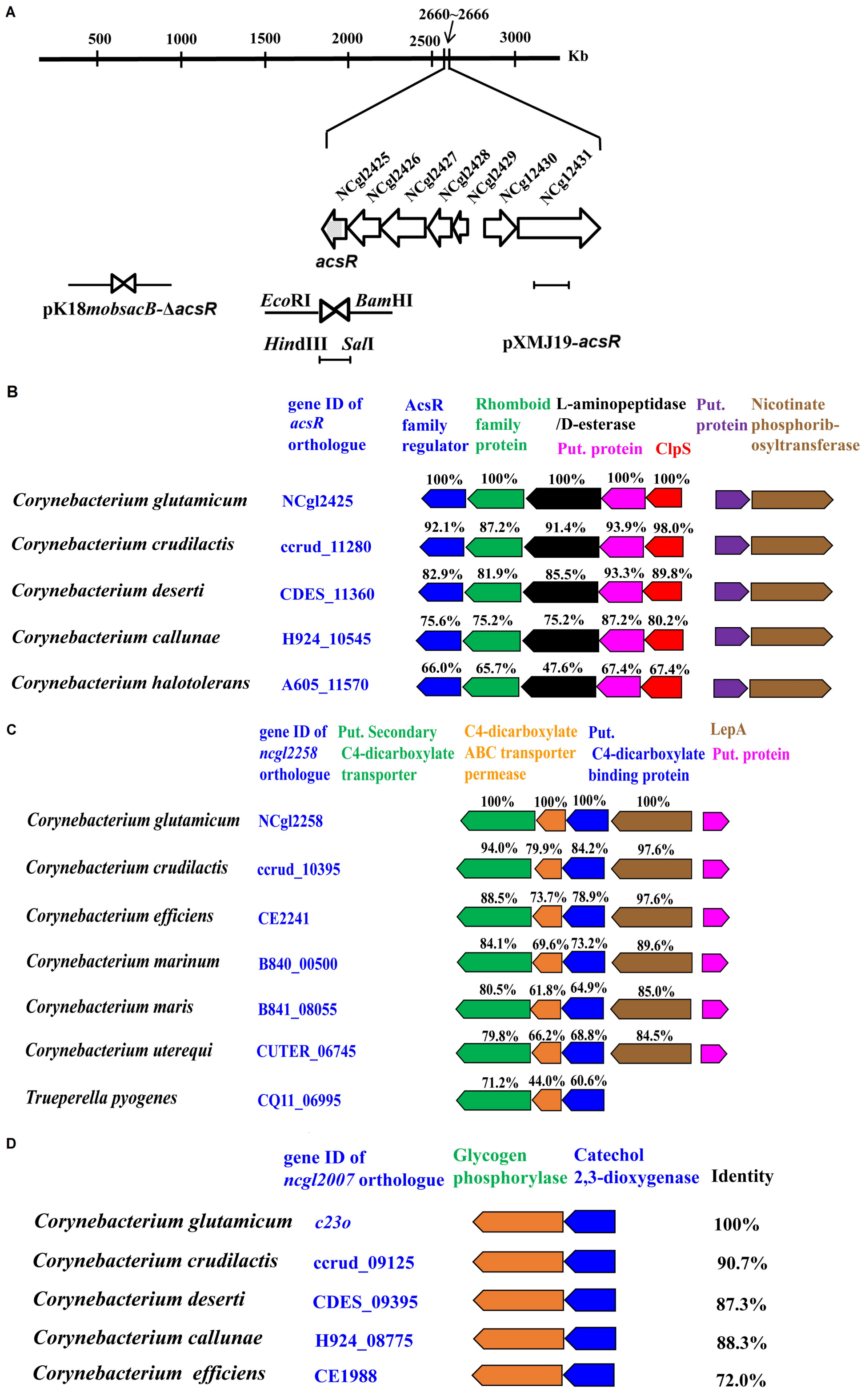

Figure S1. Comparison of the gene organization and genome locus in *C. glutamicum* and related species. (A) Detailed genetic maps of the regulatory region of AcsR. Physical map of the *clpS-ncgl2428-ncgl2427-ncgl2426-acsR* operon in *C. glutamicum* RES167 parental strain and construction of plasmids for gene disruption (pK18mobsacB derivatives) or complementation (pXMJ19 derivatives). Open reading frames (ORFs) were marked by open arrows, and the deleted regions were in grey. The restriction sites were indicated. (B) The percentage identities of the amino acid sequences to the *clpS-ncgl2428-ncgl2427-ncgl2426-acsR* from *C.*

***glutamicum* were taken from NCBI Blast. Amino acid sequence identities to the *C. glutamicum* orthologs were given in the upper column. The genomic context of *C. glutamicum clpS-ncgl2428-ncgl2427-ncgl2426-acsR* operon was extracted from microbesonline (http://microbesonline.org). (C) Comparison of the organization of the *ncgl2259-ncgl2258-ncgl2257-ncgl2256* operon in *C. glutamicum* and related species. Homologous proteins of the *ncgl2259-ncgl2258-ncgl2257-ncgl2256* operon were shown. The percentage identities of the amino acid sequences to proteins from *C. glutamicum* were taken from NCBI Blast. Amino acid sequence identities to the *C. glutamicum* proteins ortholog were given in the upper column. The genomic contexts of *C. glutamicum* proteins were extracted from microbesonline (http://microbesonline.org). (D) C23O and homologous proteins were shown. The percentage identities of the amino acid sequences to proteins from *C. glutamicum* were taken from NCBI Blast. Amino acid sequence identities to the *C. glutamicum* proteins ortholog were given in the right column. The genomic contexts of *C. glutamicum* proteins were extracted from microbesonline (http://microbesonline.org).**

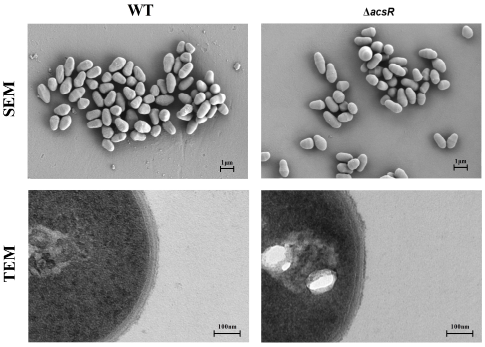

Figure S2. Cell morphology of *C. glutamicum* wild type cells (WT) and ΔacsR. Thermal field emission microscopy (SEM) pictures: the extra high tension was 2kv, magnification was ×8K, working distance was 6.8 mm, and the scale bar represented 1μm. Transmission electron microscopy (TEM) pictures: the extra high tension was 80 kv, magnification was ×30K, and the scale bar represented 100 nm. For microscopic analysis, cells were cultivated in LB medium. Cells were fixed using 3% Glutaraldehyde in Sörensen phosphate buffer.

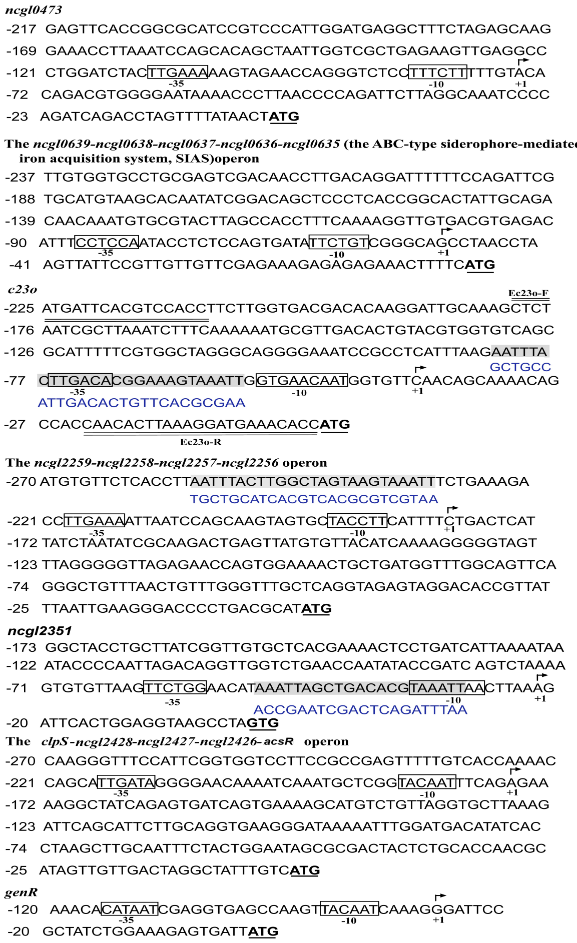

Figure S3. The promoter regions of the AcsR-regulated genes. The promoter sequences were analyzed by the Promoter Analysis and Regulon Prediction in Virtual Footprint (http://prodoric.tu-bs. de/vfp/index2.php). The deduced -35 and -10 promoter regions were boxed. Putative transcriptional start sites were indicated with arrow. The start codons of the corresponding genes were marked in bold letter and underscore. Identified AcsR binding regions were indicated with the grey font. The sequences used to replace the identified binding regions were shown in blue below the promoter sequences. A 180-bp *c23o* promoter DNA fragment was obtained with primers Ec23o-F and Ec23o-R. The sequences of primers Ec23o-F and Ec23o-R were double underlined.

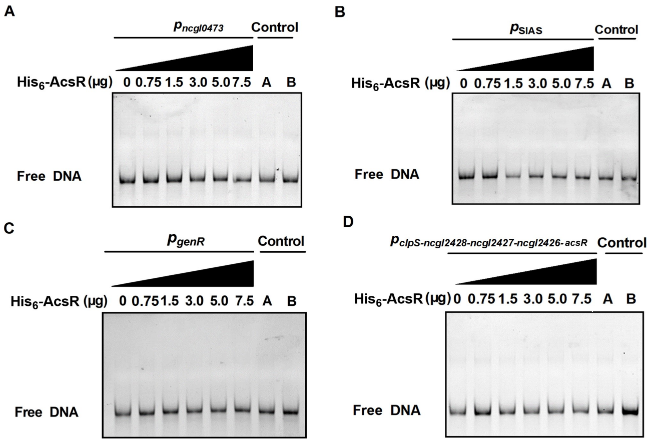

Figure S4. Electrophoretic mobility shift assays (EMSAs) were performed to analyse the interactions between His_6-AcsR and DNA fragments covering the putative promoter regions of *ncgl0473* (A), the SIAS operon (an

ABC-type siderophore-mediated iron acquisition system) (B), *genR* (C), or the *clpS-ncgl2428-ncgl2427-ncgl2426-acsR* operon (D). Increasing amounts of His$_6$-AcsR (0-7.5 μg) and 40 ng DNA fragment were used. Fragment amplified from the corresponding target gene coding region using the primers control F and control R instead of the corresponding promoter (control A) and an irrelevant protein BSA instead of His$_6$-AcsR (control B) in the binding assays were used as negative controls.

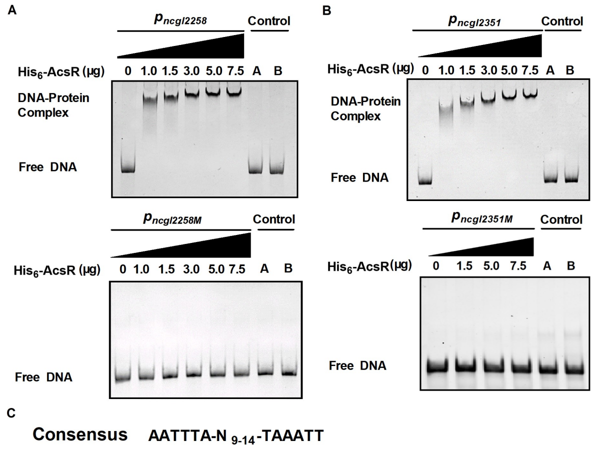

Figure S5. The promoter DNA mutations in the predicted AcsR-binding sites prevented the binding of AcsR. (A, B) The interaction between the increasing amounts of His$_6$-AcsR and the promoter DNA fragments of *ncgl2258* (A) and *ncgl2351* (B) containing or missing the proposed AcsR binding sites was performed by EMSA. (C) DNA sequences of binding sites that were verified using EMSAs were used to deduce a possible binding motif of AcsR.

	-35 -10
C. glutamicum	AGAATTTAC**TTGACA**CGGAAAGTAAATTG**GTGAACAAT**GGT-44-**ATG**
C. crudilactis	CTAATTTAG**TTGACA**TGGAAAGTAAATTG**GGGCATGAT**GTA-43-**ATG**
C. deserti	AAAATTTAG**TTGACA**CGGCAAGTAAATGG**GTGCACAAT**AGT-46-**ATG**
C. callunae	AGTATTTAC**TTGACA** TATCAAGTAAATAC **TTTTAAAGT**GAG-38-**ATG**

Figure S6. Sequence of the promoter region of *C. glutamicum c23o* aligned to putative promoter regions from other *Corynebacterium* species. Indicated were the start of *c23o* coding region (start codons black bold), putative -35 and -10 boxes (underlined and black bold), and the AcsR binding site (shaded in gray) for the *C. glutamicum c23o* gene. As shown by the alignment, also the other species possessed putative AcsR binding sites in the *c23o* upstream regions.

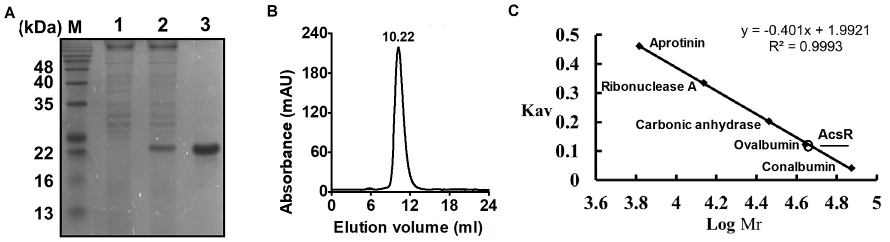

Figure S7. Purification of His$_6$-AcsR and determination of the native molecular mass by gel filtration. (A) Coomassie-stained SDS-PAGE of His$_6$-AcsR purified by Ni^{2+}-NTA chromatography. Lane M, protein markers (13 to 245 kDa); lane 1, crude extract of BL21(DE3)(pET28a-*acsR*) strain without IPTG induction; lane 2, crude extract of BL21(DE3)(pET28a-*acsR*) strain with IPTG (0.5 mM) induction; lane 3, purified His$_6$-AcsR protein; (B) Elution of native His$_6$-AcsR from size exclusion column; (C) For calibration, a premixed protein molecular mass marker containing the following proteins was used: aprotinin (6,500 Da), ribonuclease A

(13,700 Da), carbonic anhydrase (29,000 Da), ovalbumin (44,000 Da), and conalbumin (75,000 Da). V_o was determined with blue dextran (2,000 kDa).

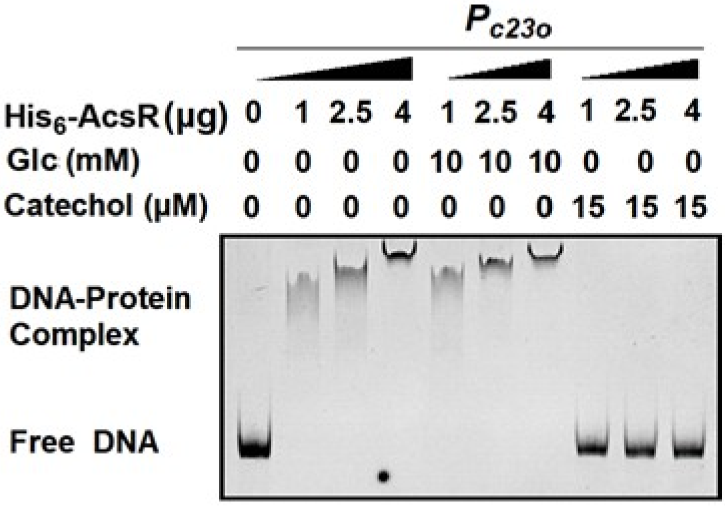

Figure S8. Inhibition of the DNA binding of AcsR by catechol. AcsR was prepared in different concentrations, and aliquots were taken for EMSAs (control). Then a final concentration of 15 µM catechol or 10 mM Glc was added to the binding reaction mixture, and aliquots were taken for EMSA.
